# Bacterial community changes in strawberry fruits (*Fragaria* × *ananassa* variety “Monterey”) from farm field to retail market stands, an indicator of postharvest contamination

**DOI:** 10.3389/fmicb.2024.1348316

**Published:** 2024-02-16

**Authors:** Gabriela N. Tenea, Pamela Reyes

**Affiliations:** Biofood and Nutraceutics Research and Development Group, Faculty of Engineering in Agricultural and Environmental Sciences, Universidad Técnica del Norte, Ibarra, Ecuador

**Keywords:** strawberries, metagenomics, 16S metabarcoding, bacterial diversity, pathogens, *Shewanella putrefaciens*

## Abstract

**Background:**

Strawberry (*Fragaria* × *ananassa*) fruits are vulnerable to bacterial contamination; some species are pathogenic and can affect human health. Comprehending the bacterial composition and diversity at different ripe stages is a key determinant of the fruit health, productivity, and quality.

**Methodology:**

An amplicon metagenomic approach on the 16S rRNA region was used to identify the bacterial diversity in exocarp of fruits collected from a farm field at two ripe stages: breaking (white, phase two) and ripe (red, phase four) and purchased from different retail market stands at ripe (red, phase four, ready-to-eat) stage. Besides, the fruit quality was assessed.

**Results:**

Strawberries carries a high microorganisms diversity, with *Pseudomonaceae, Yearsiniaceae*, and *Hafniaceae* being the most abundant families across the samples. Among the groups, *Pseudomonaceae* and *Clostridiaceae* were the most abundant families at breaking (phase two) and ripe (phase four), whereas *Yearsiniaceae, Hafniaceae, Aeromonadaceae*, and *Streptococcaceae* were the most abundant families in the market group. Although samples from group four-field and market were at the same ripe stage, the bacterial species composition was divergent. *Serratia* spp. were prevalent (above 60%) in samples collected from the market group, and *Pseudomonas* (above 70%) species were mostly found in the samples collected from the field settings regardless of the phase. Besides, *Escherichia coli* and *Salmonella enterica* were detected in the ready-to-eat samples from both the field and the market, while *Enterococcus gallinarum* was detected in the samples that originated from the market. Interestingly, *Shewanella putrefaciens* and *Shewanella profunda*, two human opportunistic pathogens, were detected in the fruits from the market only. According to alpha and beta diversity analyses, strawberry fruits displayed significant differences (*P* < 0.05) in bacterial communities within the ripe group, with the samples from the market showing the most bacterial diversity. Although we do not directly correlate the quality attributes with bacterial diversity, the results indicated a clear separation between groups according with their ripe stage and origin.

**Conclusion:**

This study provides a comprehensive framework of the bacterial diversity throughout the transition from unripe to ripe strawberries which may aid in the development of preventative measures to manage the postharvest contamination.

## Introduction

Strawberries (*Fragaria* × *ananassa*) have a high labor cost and value and are mainly consumed as fresh fruit. To maximize consumer acceptance, strawberries must be picked at the proper time before they stop ripening and have a limited shelf life (up to 3 days at room temperature and 7 days at 4°C) (Abdelfattah et al., 2016). The capacity to keep fruits fresh is significantly hampered by postharvest degradation by microbes (Sharma et al., 2009). Given the initial investment made in crop production and the control of pathogens and pests in the field before harvest, the financial losses brought on by storage damages are significant. Strawberries are vulnerable to several fungus and bacterium contaminations, making fungicide treatments compulsory (Abdelfattah et al., 2016). The options for controlling postharvest contamination have also been constrained due to concerns about human health (Droby and Wisniewski, 2018). Applications of fungicides after harvest have been banned or severely regulated in several nations (National Pesticide Information Center, 2023). As a result, efforts are being made to develop alternative management strategies to decrease fungicide use, lower environmental concerns, and raise customer confidence in the food safety. The postharvest environment has an advantage over field settings where environmental factors are more stable, and parameters can, to some extent, be changed to promote the survival of biocontrol agents. The difficulty is that new products must not only be secure but also efficient and affordable (Poleatewich et al., 2023).

In the case of fresh, ready-to-eat fruits or minimally processed fruits, the microbiomes present on the surface (exocarp) have been thoroughly studied, primarily to detect pathogenic bacteria and their potential connection with human disease outbreaks (Jackson et al., 2015; Wassermann et al., 2019). The detected microbes have been linked to postharvest procedures as well as via contact with contaminated human hands, rinse water, improper transportation, and storage conditions (Heaton and Jones, 2008; Leff and Fierer, 2013). Potential human pathogens were found in fruits and vegetables sold in Delhi-NCR retail stores (Saksena et al., 2020). Besides, *Escherichia coli* O157:H7 survived on the surface and within the pulp of strawberries during production and postharvest handling (Yu et al., 2001). Although studies of the strawberry bacterial and fungal communities have been conducted in both plants and fruits (Jensen B. et al., 2013; Abdelfattah et al., 2015), still limited knowledge exists related to the bacterial diversity in ready-to-eat strawberries originated from farm settings and open air markets.

The microbiome might be also impacted by the farming conditions and continental location (Abdelfattah et al., 2021). Thus, the strawberry-associated microbiome is species-specific impacted by management and connected to the phyllosphere and rhizosphere plant-soil feedback (De Tender et al., 2016). In addition, fruit-associated microbiome is linked to their shelf-life (Kusstatscher et al., 2020).

In Ecuador, strawberry production has become one of the main economic axes of several indigenous communities (Huaycopungo community, San Rafael parish), and it currently constitutes the second source of income in the region after reed crafts. Only 12% of strawberry growers are committed to exporting their products. The strawberry farming settings are located at a height of 2,300–2,500 m, where the soil is mainly volcanic, the temperature varies between 18 and 25°C during the day and between 8 and 13°C during the night with a relative humidity of 60%. Besides, due to a lack of training on the use of agrochemicals, farmers handle these fruits without taking the appropriate safety measures, which adds to the region empiricism in managing strawberry production (Asociación de Municipalidades, Ecuador, 2021). Using conventional targeted microbiological methods, we detected high content of *Enterobacteriaceae* in strawberry fruits purchased from the local market (Tenea et al., 2023). Among them several isolates showed multi-drug resistance. Many *Enterobacteriaceae* members are among the most powerful and common pathogens in clinical settings, their resistance to antibiotics has also become a major concern as the efficacy of antimicrobial treatments is reduced (Zurita et al., 2020).

In this study, we propose to use the 16S rRNA-based metagenomic approach to track uncommon and unforeseen pathogens and non-pathogenic bacteria in strawberry fruits collected from Northern Ecuador farm field at two ripeness stages: breaking (white, phase two) and ripe (ready-to-eat, phase four) and fruits purchased from a local retail market (ready-to-eat, phase four). In addition, standard procedures were used to assess the fruit's physicochemical parameters (pH, total titratable acidity, and total soluble solids) and active substances (antioxidant capacity, total polyphenols, and vitamin C content) to determine whether any differences existed between the groups and their origin and ripe stage. The diversity of fruit associated bacteria throughout the transition from the field to the market may help to identify the critical points in the process chain, may offer a way to enhance the fruit safety and quality as well as may contribute to further develop preventative measures against postharvest damages, potentially lowering both food losses and human illness.

## Materials and methods

### Sample collection, experimental design, and processing

**Figure 1:**
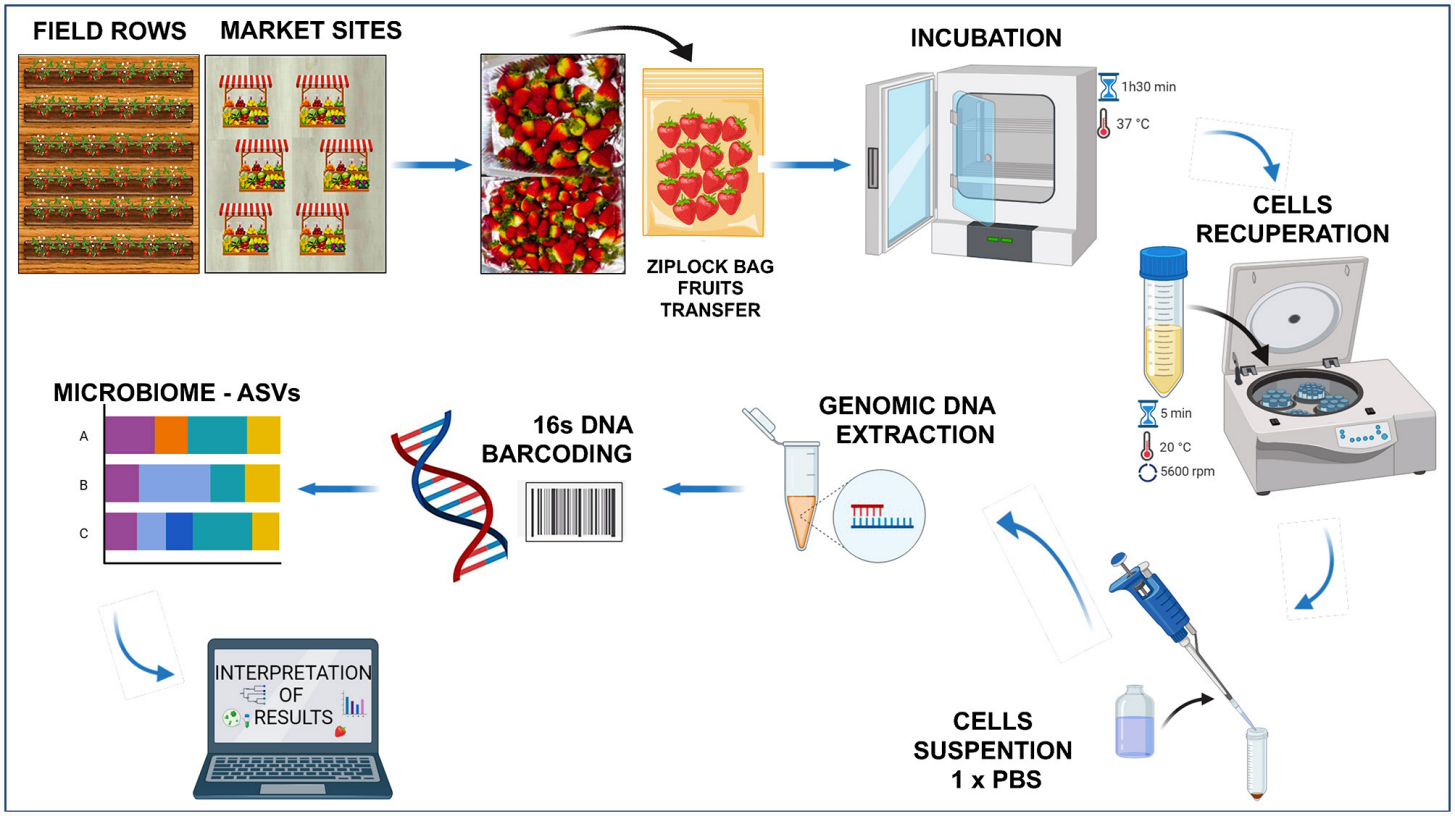
Strawberry processing workflow (Created with BioRender.com).

In this study, *Fragaria* × *ananassa* variety “Monterey,” an ever-bearing strawberry that produces fruits anytime in contrast to regular cultivars, was used. Strawberries were obtained from both farming settings (local producer geographical coordinates 0°14′00″N 78°16′00″O) and retail market stands of Ibarra city (capital of Imbabura Province, Northern Ecuador). The fruits with no visible damages: 15 fruits per row × six field rows × two ripeness stages × three repetitions (total 540 fruits) were collected randomly from the field at two ripeness stages: breaking (white; phase two) and ripe (ready-to-eat; phase four) (Zhang et al., 2011) during February–October 2023. Likewise, from the retail market, the fruits were purchased from six different stands: 15 fruits × *six* stands × *three* repetitions (total 270 fruits) at the ripe phase four (ready-to-eat). The fruits were transported to the laboratory and placed in Ziplock bags containing 150 ml sterile peptone water (0.1%), and gently mixed manually every 30 min during incubation for 1.30 h at 37°C. The cells surrounding the fruit skin (exocarp) were collected by centrifugation at 8,000 × g for 5 min, recovered in 1 × PBS and stored at 4°C before analysis. Figure 1 shows an overview of the workflow process.

### 16s rRNA gene metagenomics

#### DNA extraction, library construction, and sequencing

Genomic DNA was isolated using a commercial column-based ZymoBIOMICS DNA miniprep kit following the instructions of the manufacturer (#4300, Zymo Research, California, US). DNA quality and integrity were determined using a *PicoGreen* dsDNA Quantitation Reagent (# 7581, Promega, USA). Metagenomic sequencing was performed on an Illumina Novaseq platform (paired-end 150 bp reads, Illumina, USA). The 16S rRNA V3-V4 region was amplified with the bacterial primers 341F (5′-CCTACGGNGGCWGCAG-3′) and 805R (5′-GACTACHVGGGTATCTAATCC-3′) (Klindworth et al., 2013). All polymerase chain reaction (PCR) reactions involved the KAPA HiFi HotStart ReadyMix (Sigma Aldrich, USA). A washing step using magnetic beads was applied to purify the 16S, V3, and V4 amplicons from free primers and primer dimer species. Using the full complement of Nextera XT indices, up to 96 libraries were pooled for sequencing. Library preparation consisted of adding indices to each end of the previously obtained amplicons. The second wash was performed using magnetic beads to clean the final library. Finally, the purified libraries were quantified and qualified to determine their suitability for sequencing. Two different categories of negative controls were used: (1) All DNA extraction and subsequent technique stages contained a blank extraction control. There was no input data for this empty control. (2) A control library that used DNA-free water as the input for library formation and subsequent sequencing instead of using the extraction process (Díaz et al., 2023).

### Data processing and analysis

The metagenomics workflow is a secondary analysis option built into the MiSeq Reporter (on-system software) or available on BaseSpace (cloud-based software; Illumina, USA). FASTq files were subjected to a quality and filtering process to guarantee taxonomic classification. The workflow is shown in Supplementary Figure 1. The QIIME v.2 (Quantitative Insights into Microbial Ecology) pipeline (Bolyen et al., 2019), version 2023.5, was used for marker gene-based microbiome sequencing data analysis. Sequences were denoised using the denoise wrapper (Reeder and Knight, 2010), and the 16S region was extracted. Removal of noise (denoising) and grouping of sequences (clustering) were performed with DADA2 (Callahan et al., 2020). DADA2 replaces the traditional “OTU selection” step in amplicon sequencing workflows, instead producing higher-resolution tables of amplicon sequence variables (ASVs) (Alishum, 2021). Chimeric sequences were identified and filtered using USEARCH 6.1 (Edgar, 2010). Moreover, a taxonomy is assigned to the sequences. This step allows to evaluate which organisms are represented by the ASVs. Further, the query sequences (ASVs) were compared with a reference database of sequences with known taxonomic composition. Simply finding the closest alignment is not enough because other sequences that are equally close or almost as close matches may have different taxonomic annotations; therefore, a taxonomy classifier was used to determine the closest taxonomic affiliation with some degree of confidence or consensus, depending on the alignment and k-mer frequencies. In this specific case, a Bayesian classifier was used against the SILVA v.132 reference taxonomy database for bacteria (Quast et al., 2012). Sequences belonging to chloroplasts, mitochondria, and eukaryotes were removed.

### Rarefaction curves

Rarefaction curves were used to evaluate the diversity of species or biological characteristics present in a sample and how this diversity varies depending on the number of sequences (reads) analyzed (Kandlikar et al., 2018). These curves are useful for understanding how sequencing depth affects the ability to capture the full diversity of a sample.

### Alpha diversity analysis

Alpha diversity metrics are typically weighted by the abundance at which individual microbes are observed. The analysis involves calculating alpha diversity indices for each sample and comparing indices values between different samples or groups to understand how species composition varies within a specific group or sample. The following metrics were calculated: Shannon diversity index (used to estimate the diversity of species within the groups) (Krebs, 2014); Evenness/Pielou uniformity is an index that measures diversity along with species richness. The results were visualized in boxplot figures.

### Beta diversity analysis

Beta diversity analysis was used to determine the differences between all the samples from each group (phase two, phase four, and market). The sequence read data were organized at the lowest taxonomic level and normalized for sampling depth, square root transformed, and a resemblance matrix created by calculating Bray-Curtis coefficients (Lozupone et al., 2007). The statistical significance of the detected differences was evaluated using weighted and unweighted UniFrac distance matrices and 999 Monte Carlo permutations. Principal coordinates analysis (PCoA), as implemented in QIIME 2.0, was used to relate the bacterial community composition to the sample group. The summary of beta diversity relationships can be visualized in two- or three-dimensional scatter diagrams. A significance level of 0.05 was considered for all statistical procedures.

### Statistical significance tests

For alpha diversity, the results were compared using a nonparametric Kruskal–Wallis test, or one-way ANOVA on ranks, to test whether samples originate from the same distribution. The null hypothesis of the test is that the mean ranks of the groups are the same. In the case of beta diversity, the PERMANOVA was run with the default settings and 999 permutations, and PERMANOVA-derived significance values were considered significant when *P* < 0.01 (Anderson, 2017). In addition, the ANOSIM (analysis of similarities) was used for testing the hypothesis that there is no difference between two or more groups of samples based on permutation testing of similarities between and within groups. The *R*-test statistic was used to test if there are no differences between the groups under the null hypothesis (Yinglin et al., 2018). Besides, the Analysis of Microbiome Composition (ANCOM) (Mandal et al., 2015) was used to identify the taxa that have significantly differential abundance to infer the microbial absolute abundances. It considers the compositionality of microbiome data using a centered log ratio (CLR) transformation. For a given taxon, the output *W* statistic represents the number of CLR-transformed models where the taxon is differentially abundant with the variable of interest. The higher the value of *W*, the more likely it is that the taxon is differentially abundant.

### Fruit quality evaluation

Physicochemical characterization, total polyphenol content (TPC) estimation, ascorbic acid content (AAC), and antioxidant (AOX) activity were determined in all samples collected from the farming field (phases two and four) as well as from the market (phase four). Using phenolphthalein as an indicator, the total titratable acidity (TTA) was measured by titrating 25 ml of pulp juice obtained with 0.1 N NaOH [Association of Official Analytical Chemists (AOAC), 2003]. Results were expressed as a percentage of ascorbic acid per 100 ml of juice. The pH was determined using an electrode immersion pH meter (S210, Mettler Toledo, Columbus, OH, USA). Total soluble solids (TSS) content was determined using a digital refractometer [Association of Official Analytical Chemists (AOAC), 2003]. For TPC estimation, the Folin-Ciocalteu method with gallic acid (Sigma-Aldrich Co. LLC, Saint Louis, MO, USA) as standard was used as previously described (Singleton et al., 1999). Vitamin C (reduced ascorbic acid) content was determined following a standard 2,6-dichloroindophenol titrimetric method [Association of Official Analytical Chemists (AOAC), 2003]. The DPPH (1, 1-diphenyl-2-picryl-hydrazyl, Sigma-Aldrich Co. LLC, Saint Louis, MO, USA) radical scavenging activity was determined as described (Rojas-Ocampo et al., 2021).

## Results and discussion

### Alpha diversity and evenness

**Figure 2:**
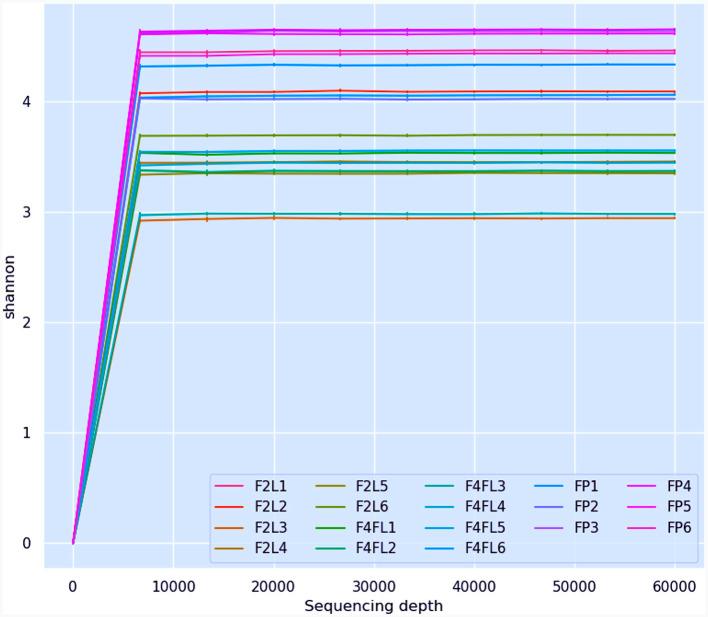
Illustration of rarefaction curves determined for all strawberry samples (sequencing depth 4,000). F2L1–F2L6: fruits collected from agricultural field at breaking (white) ripe phase two; F4L1–F4L6-fruits collected from agricultural field, ripe phase four; FP1–FP6: fruits purchased from market, ripe phase four.

**Figure 3:**
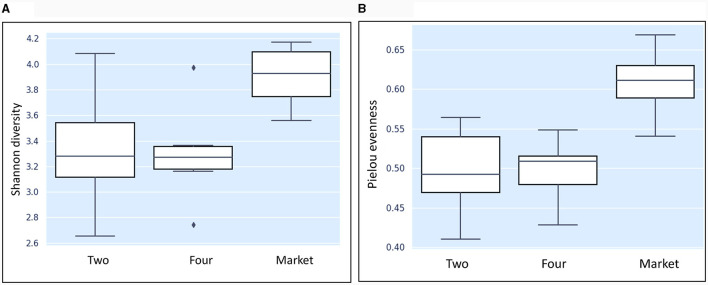
Boxplot results of the nonparametric Krustal-Wallis comparing the Shannon diversity index **(A)** and evenness **(B)** among the groups. Two: group of fruits collected from the agricultural field, breaking phase two; four-group of fruits collected from the agricultural field phase four; market: group of fruits purchased from the market phase four.

In this study, the bacterial community composition of strawberries at both unripe and ripe stages collected from farm field settings and market stands was evaluated via metagenomic analysis. The characteristics of the input non-chimeric reads obtained for each sample are described in Supplementary Table 1. The sequenced negative control did not yield any bacterial sequence. Table 1 shows the characteristics of ASVs filtered across the samples. The rarefaction analysis indicated that the sequencing depth of 60,000 was sufficient to capture most taxa present in the samples (Figure 2). Examining the curve, it can be observed how it flattens out when additional readings are gathered. The alpha diversity analysis indicated that strawberry fruits originating from the market presented the most diverse bacterial community, followed by fruits originating from the agricultural field (Table 1). We observed a slight decrease in the Shannon index in the samples that originated from the field (Table 1). According to the current study, the greater diversity of microorganisms in fruits purchased from the market may be linked to the environment and storage conditions rather than their maturity stage. A comparison between bacterial diversity in each group and their evenness (Pielou uniformity) is shown in Figures 3A, B. However, a statistically significant (*P* = 0.016) difference was observed between group four (field setting) and the market group, although the fruits were at the same ripeness stage. No statistical difference (*P* = 0.087) was noted when comparing samples from phases two and four, indicating that at the field level, the fruits shared similar bacterial diversity, while out of the field setting, the diversity increased (Supplementary Table 2). Besides, minor taxa (relative abundances < 1%) included several bacterial species that were detected in some samples but absent (relative abundance of 0%) in others, suggesting that they were independent. This result was not unexpected, as we showed previously using conventional targeted bacteriological analysis that some bacteria were prevalent in fruits originating from the market rather than the field (Tenea et al., 2023). In a recent metabarcoding study of pear fruits grown in conventional and organic fields, it has been shown that postharvest fruit quality and decay could be potentially attributed to the variation in the microbial community during storage (Gao et al., 2023).

**Table 1:** Sequences characteristics and Shannon diversity index.

**Sample ID**	**Phase**	**Sequence counts**	**Filtered counts**	**Shannon diversity index**	**Total species-level taxonomic categories identified**
F2L1	Two	108,643	108,494	4.456	779
F2L2	Two	137,660	137,478	4.082	691
F2L3	Two	161,627	161,344	2.945	761
F2L4	Two	126,432	126,261	3.456	707
F2L5	Two	157,033	156,776	3.351	816
F2L6	Two	144,945	144,769	3.689	944
F4L1	Four	102,942	102,854	3.532	859
F4L2	Four	90,508	90,453	3.364	628
F4L3	Four	105,951	105,884	2.979	741
F4L4	Four	155,305	155,110	3.438	513
F4L5	Four	140,674	140,594	3.554	504
F4L6	Four	118,178	115,792	4.056	607
FP1	Market	76,428	75,288	4.326	1,178
FP2	Market	71,147	69,568	4.018	675
FP3	Market	110,743	107,460	4.633	807
FP4	Market	150,194	149,623	4.647	580
FP5	Market	177,833	176,438	4.609	505
FP6	Market	202,471	192,328	4.442	705

F2L1–F2L6: fruits collected from the agricultural field at breaking (white) ripe phase two; F4L1–F4L6: fruits collected from the agricultural field, ripe phase four; FP1–FP6: fruits purchased from the market, ripe phase four.

### Beta diversity

**Figure 4:**
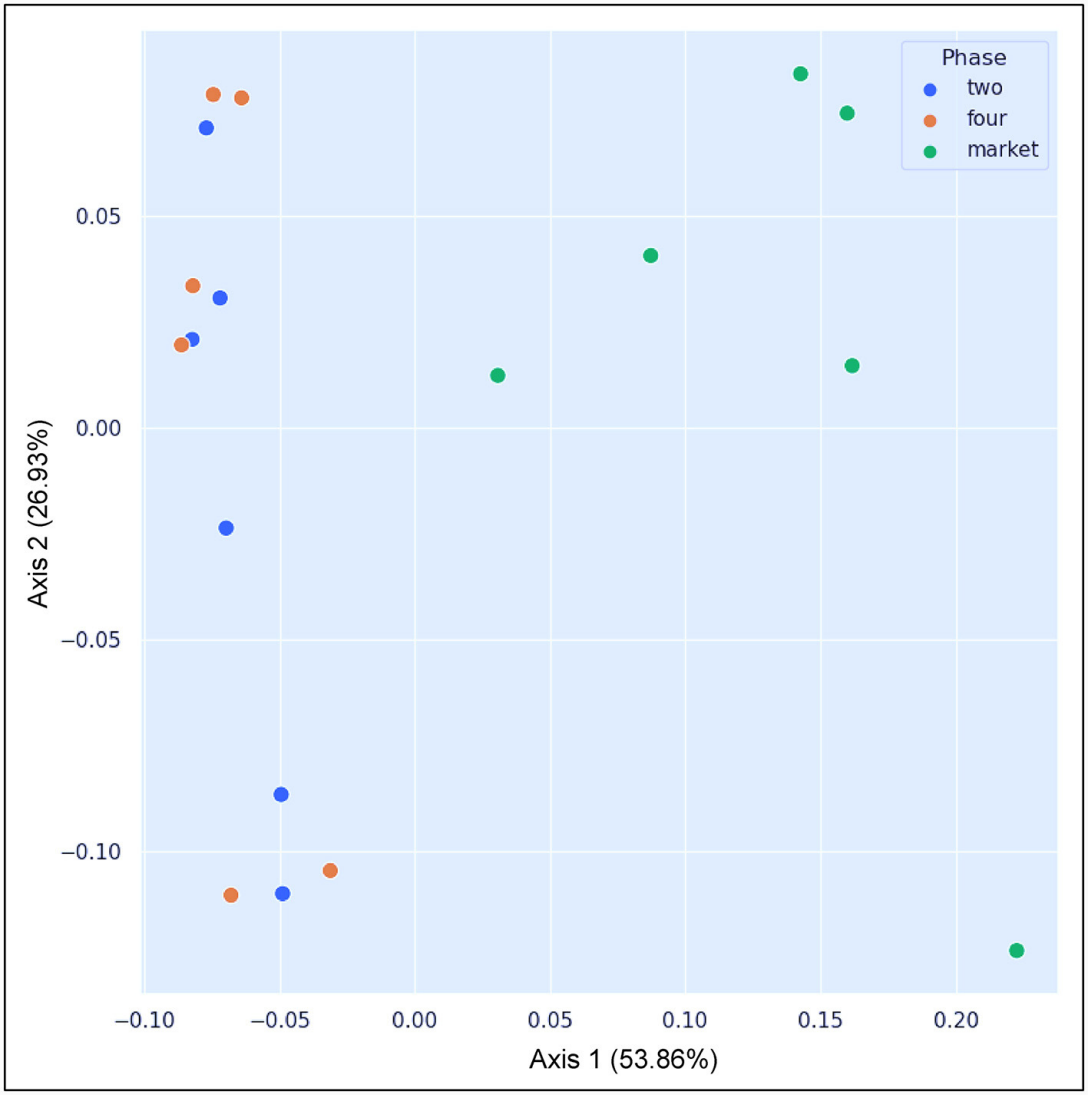
PCoA plot showing the results of the beta diversity analysis using the weighted UniFrac metrics. Colored circles were used to indicate the samples that belong to same group: blue: samples collected from agricultural field settings, breaking phase two; orange: samples collected from the agricultural field setting, ripe phase four; green: samples collected from the market, ripe phase four.

The market group samples created a cluster that is distinct from the field samples regardless of their ripe stage, as can be seen in the PCoA map for the abundance weighted UniFrac distance (Figure 4). The variable F1 (Axis 1) explained 53.86% of the total variance by loading the samples from the market in the positive direction, while F2 (Axis 2) explained 26.93% by loading the samples from field settings from phases 2 and 4. Likewise, microbial inter-group diversity (PERMANOVA) based on weighted UniFrac metrics revealed significant differences (*P* = 0.05) between the bacterial communities present on fruits originating from the field (phase four) and the market (phase four), while no significant differences were observed between phases 2 and 4, which originated from the same field (Table 2). A larger pseudo-F value suggests a larger group separation. Moreover, based on the similarity analysis (ANOSIM) results, R values between group four and the market as well as group two and the market were close to “1.0” (0.71 and 0.72, respectively), suggesting dissimilarity between groups, while the R values close to “0” (-0.085) suggest a uniformity distribution between group two and four from the field. This may be caused by the varied abundances of several significant bacterial taxa in the market (e.g., *Gluconobacter, Frateuria*), as well as the presence or absence of those taxa in the samples collected from the field (see below in taxonomy). Research on beta-diversity revealed that the microbiomes of 15 açai fruit samples, collected from dry land farming areas and native cultures grown in low floodplains with varying levels of spontaneous decay during postharvest, were highly similar (Moura et al., 2018). It is important to highlight that in the current study, the fruits were collected from the field where traditional practices (fungicidal treatments) were used; nonetheless, the bacterial diversity varies considerably when comparing both field and market groups; thus, the fruit contamination should not be neglected once collected from the field. The bacterial communities have the capacity to change considerably, and the increase in several harmful pathogens makes the safety of consumers uncertain. Therefore, effective decontamination approaches are necessary for improving the microbial safety of fruits and minimizing health hazards.

**Table 2:** Pairwise PERMANOVA results according to weighted UniFrac metrics (β-diversity).

**Group 1**	**Group 2**	^*^Pseudo-*F*	***p*-value**	***q*-value**
Four (field)	Market	9.674	0.003	0.005
Four (field)	Two (field)	0.320	0.760	0.760
Market	Two (field)	10.655	0.003	0.005

The significance was determined through 999 Monte Carlo permutations the values were considered significant when p < 0.05.

^*^Pseudo-F value determined according to Lattin et al. (2003).

### Bacterial community composition

**Figure 5:**
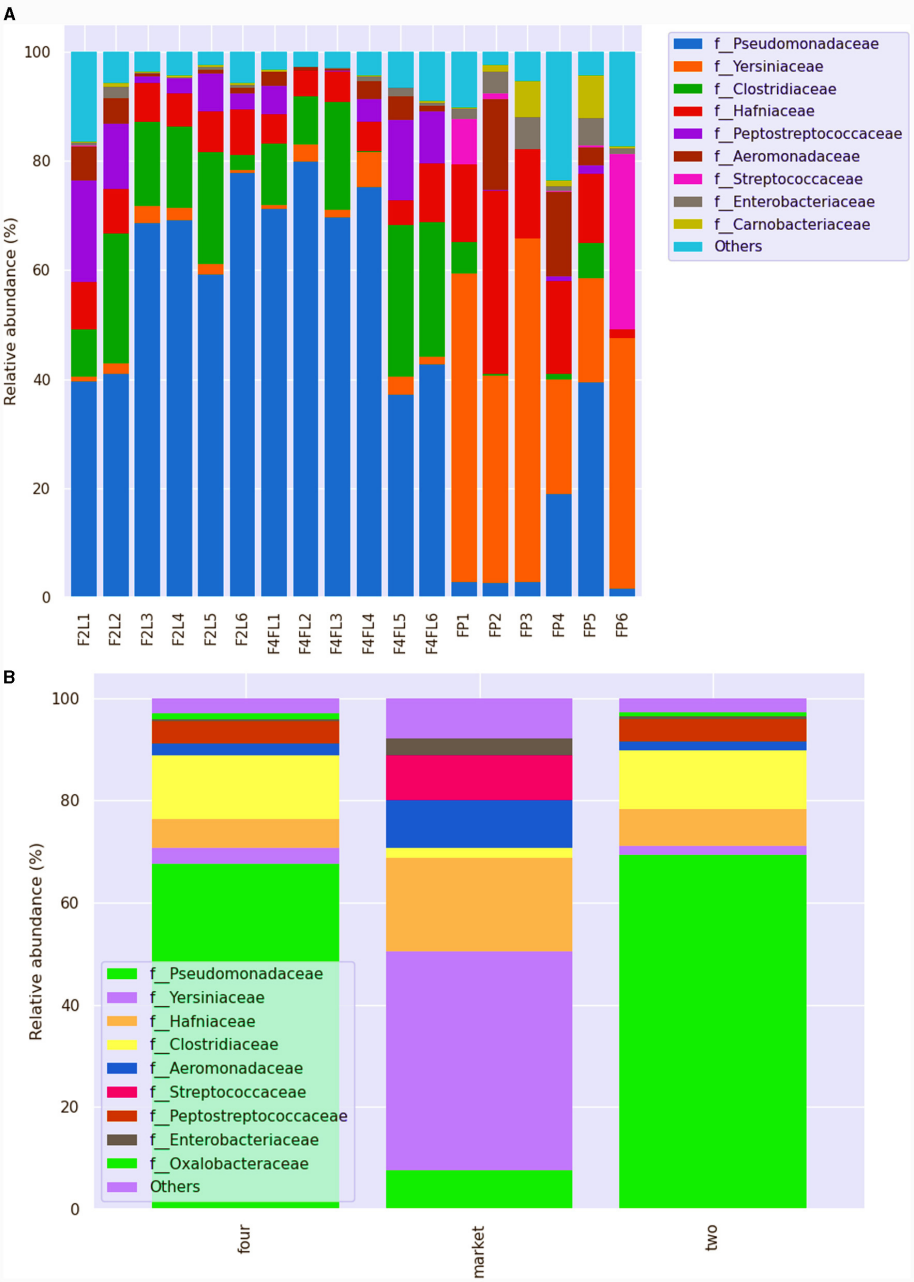
Relative abundance of different bacteria families across the samples **(A)** and among the groups **(B)** identified in strawberries. This bar chart shows the relative abundance of the top 10 classification results within each taxonomic level. F2L1–F2L6: fruits collected from the agricultural field at breaking (white) ripe phase two; F4L1–F4L6-fruits collected from the agricultural field, ripe phase four; FP1–FP6: fruits purchased from the market, ripe phase four. The “Other” category in this sum of all classifications with <0.15% abundance.

Fruits are naturally susceptible to contamination from the field, water used to clean the surface, equipment contact, and storage conditions at the postharvest stage (Balali et al., 2020). The microbial community of ready-to-eat fruit undergoes significant changes during postharvest storage, with diversity and abundance varying with storage temperature and after the application of a postharvest fungicide (Umar et al., 2023). The main taxa of each sample or group in each taxonomic rank (phylum, class, order, family, genus, species) were shown by the results of the taxonomic annotation. However, the relative abundance of taxa was represented by a histogram for each sample and classification level. At the phylum level, Proteobacteria, Firmicutes, Bacteroidota, Fusobacteria, and Actinobacteria were the most abundant across the samples. At the family level, all samples showed a high diversity of microorganisms, with *Pseudomonaceae, Yearsiniaceae*, and *Hafniaceae* being the most abundant across the samples (Figure 5A). Among the groups, *Pseudomonaceae* and *Clostridiaceae* were the most abundant families in fruits at both unripe (phase two) and ripe (phase four), whereas *Yearsiniaceae, Hafniaceae, Aeromonadaceae*, and *Streptococcaceae* were the most abundant families in the market group (phase four; Figure 5B). These families were prevalent in grapes or melon pulp, as shown in previous studies (Zarraonaindia et al., 2015; Glassner et al., 2018). At the genus level, a high proportion of *Pseudomonas* was found in samples collected from farming field settings (43%−97%) regardless of the phase, while the samples collected from the market showed a high proportion of *Serratia* (12%−65%). Based on the results, the proportion of the genus was group- and sample-dependent. In a recent metagenomic study on different tropical fruit (papaya, guava) surfaces, it was shown that the bacterial relative abundance is dependent on the ripe stage as well as on fruit variety (Vermote et al., 2022). Additionally, metagenomic analysis showed that *Pseudomonas* and *Ralstonia* were the most common taxa that survived the processing treatments of apple fruits, suggesting that these taxa need to be closely monitored as they may have an impact on the quality of the fruit (Wicaksono et al., 2022). Although in lower abundance, several microorganisms from the *Staphylococcus* (0.65%), *Corynebacterium* (< 0.5%), and *Dysgonomonas* (11.99%) genera were detected in the strawberry samples collected from the market. These variations could be attributed to differences in the pre- and postharvest environments since the fruits are more vulnerable to harmful bacterial growth in the market due to temperature and improper storage conditions. Raw sequence data were deposited in the National Collection of Biotechnology Information under accession number PRJNA1039505 (October 14, 2023).

### Taxonomy at the species level indicates the presence of potential human pathogens

**Figure 6:**
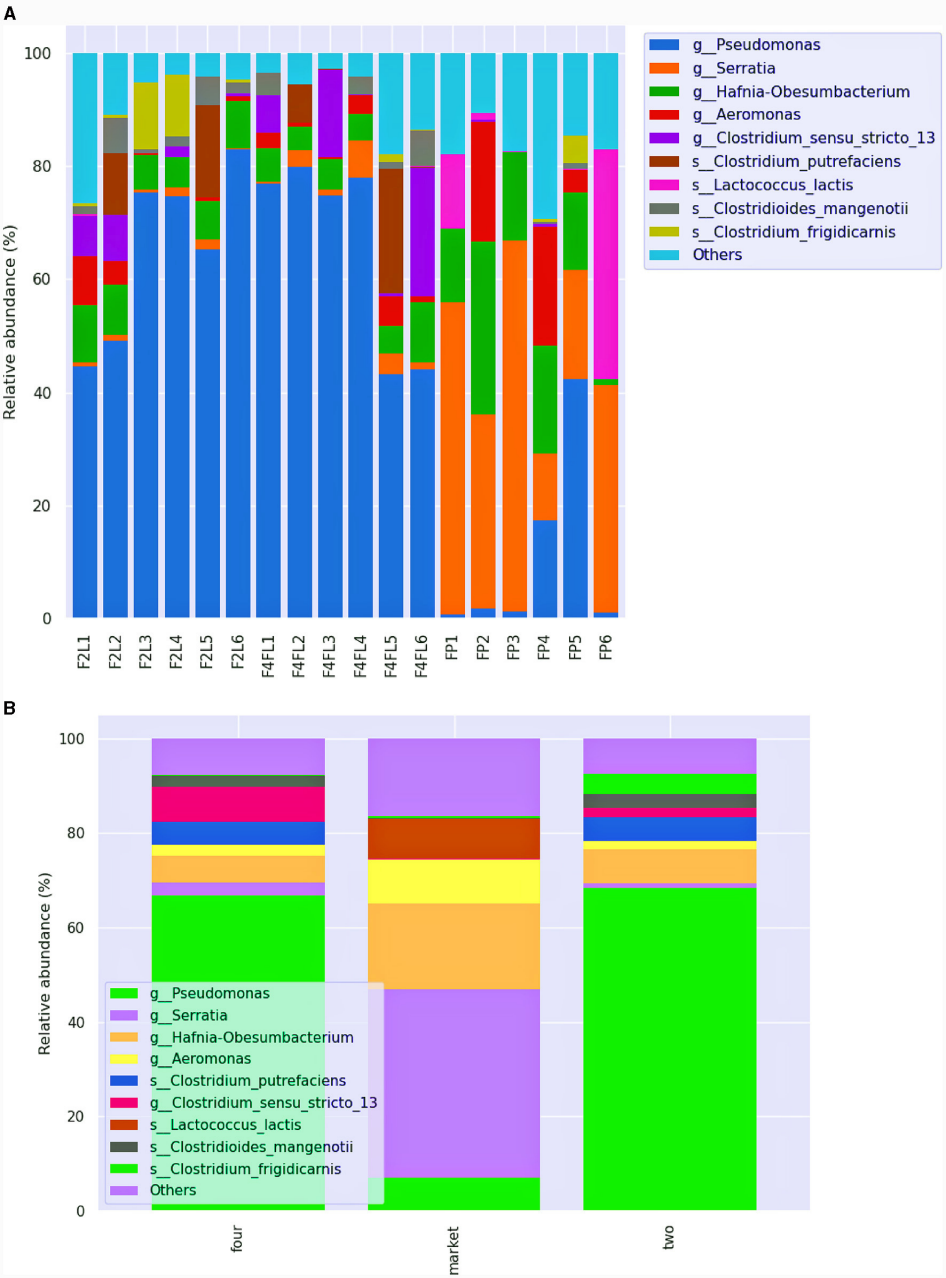
Relative abundance of different bacteria species across the samples **(A)** and among the groups **(B)** identified in strawberries. This bar chart shows the relative abundance of the top 10 classification results within each taxonomic level. F2L1–F2L6: fruits collected from the agricultural field at breaking (white) ripe phase two; F4L1–F4L6: fruits collected from the agricultural field, ripe phase four; FP1–FP6: fruits purchased from the market, ripe phase four. The “Other” category in this sum of all classifications with <0.15% abundance.

Although samples from group four and the market were in the same stage of maturity, the bacterial species composition was divergent. *Serratia* spp. were prevalent (above 60%) in samples collected from the market group, while *Pseudomonas* (above 70%) species were mostly found in the samples collected from the field settings, regardless of the phase (Figures 6A, B). Based on ANCOM analysis, we found that the most differentially abundant taxa were *Frateuria, Gluconobacter, Yersinia, Lactococcus*, and *Lactobacillus* (Supplementary Table 3). *Serratia* spp. are opportunistic pathogens of the *Enterobacteriaceae* family, with *Serratia marcescens* and *Serratia liquefaciens* causing various diseases including conjunctivitis, cystitis in humans and bloodstream infections (Hume and Willcox, 2004; Liu et al., 2004); those species, although in lower abundance (< 0.5%), were detected in fruits originated from the market only. Recent research associated the presence of *Serratia* and *Acetobacter* with diseased strawberry fruits of “Mara des Bois” and “White ananas” cultivars (Olimi et al., 2022). Besides, *S. marcescens* was found responsible for black rot disease in orange fruit in Bangladesh (Hasan et al., 2020). Furthermore, we found that *Frateuria aurantia* was the most prevalent (0.96%) among Proteobacteria in the samples that were taken from the market. Based on a comparative microbiome study of peaches, *F. aurantia* was associated with mummified fruits (Jo et al., 2020). Certain strains of *Gluconobacter* have been shown to contribute to fruit postharvest losses through browning and rot. They have frequently been found isolated from rotting pears and apples (Gao et al., 2023). Besides, *Escherichia* spp. and *Salmonella enterica* were detected in the ready-to-eat samples from both the field and the market, while *Enterococcus gallinarum* was detected in the samples that originated from the market. These results are consistent with our recent study, which found that strawberries from low-cost retail markets had higher levels of *Enterobacteriaceae* in addition to yeasts and molds (Tenea et al., 2023). Early studies on fruit collected from different agricultural conditions indicate the presence of opportunistic human pathogens along with plant pathogens, as well as several mycotoxin markers (Jensen M. M. et al., 2013). Early research indicated the presence of potential pathogenic enteric bacteria of the genera *Escherichia, Enterobacter, Raoultella, Klebsiella, Pantoea, Shigella, Citrobacter*, and *Cronobacter* in postharvest strawberries in Queensland, Australia's Sunshine Coast region, indicating the need of developing control strategies for those emerging pathogens (Kurtböke et al., 2016). Likewise, in our previous research, several clones from *Enterobacteriaceae*, isolated from strawberries were multidrug resistant (Tenea et al., 2023). Among the most resistant clones, the 16S rRNA sequencing results confirmed some taxa such as *S. liquefaciens, E. coli, Raoultella terrigena, Enterococcus faecalis, Klebsiella aerogenes*, and *Erwinia aphidicola*. Besides, several plant pathogens, such as *Erwinia rhapontici, Tatumella terrea*, and *Rahnella bruchi*, were detected in fruits from the market and, to a lesser extent, in fruits at the ripe stage four collected from the field. In addition, these pathogens have been connected to disease outbreaks in a range of fruit crops, and they affect fruit quality (Umar et al., 2023). For example, using metagenomic analysis, *Tatumella* was detected as the principal cause of mango resin canal discoloration, a disease that has a significant negative impact on the fruit's quality, undermines customer confidence, and decreases sales, although its biological origin is still unknown (Umar et al., 2023). *Erwinia* spp. and *Pseudomonas* spp., among other bacteria, inflict serious harm on fruits and vegetables (Li et al., 2023). Many serious postharvest diseases develop fast, severely deteriorating the product and sometimes ruining the entire fruits batch. Besides, a phytopathogen responsible for acute oak decline (Petfifor et al., 2020), *Brenneria goodwinii*, was detected in samples FP2 and FP5 that originated from the market. Interestingly, although at lower relative abundance (< 0.80%), *Shewanella putrefaciens* and *Shewanella profunda*, members of the *Gammaproteobacteria* class known as fish pathogens (Jayalekshmi et al., 2022), were detected in the fruits from stands FP4 and FP5 only. These bacteria are uncommon, human-pathogenic opportunistic organisms most linked to skin and soft tissue and intra-abdominal infections (Müller et al., 2023). Furthermore, all market group samples showed a lower abundance of *Shewanella amazonensis* (0.04%). This is an aerobic bacteria that was found in water of Amazon River; however, we speculate that it could be acquired during plant watering as usually farmers apply water river to the field. In a recent literature review, *S. putrefaciens* has been reported in association with more severe diseases such as pneumonia, intracerebral and ocular infections, and endocarditis (Müller et al., 2023). In addition, *S. putrefaciens* infection caused bacteremia (Benaissa et al., 2021). Instead, spore-forming bacteria such as *Clostridium putrefaciens*, with a relative abundance varying from 6.9 to 21.95%, were detected across the samples and originated from the field. *Clostridium* species are a component of the microbiota of plant raw materials, which are used as ingredients in many diets, and thus are the major bacterial groups responsible for food deterioration (Lorenzo et al., 2018). A facultative aerobe, gram-negative bacteria known as *Dysgonomonas capnocytophagoides*, was found in every group. According to recent research, this bacteria has a high level of intrinsic antibiotic resistance and is a human pathogen (Schall et al., 2021). Deguenon et al. (2019) reported that *Dysgonomonas* was identified at the genus level in black blowflies (Diptera). Although we are unable to fully understand the influence on human health at this level, the newly discovered species should not be disregarded in terms of safety.

### Taxonomic classification indicates the presence of lactic acid bacteria in fruits originating from the market

**Figure 7:**
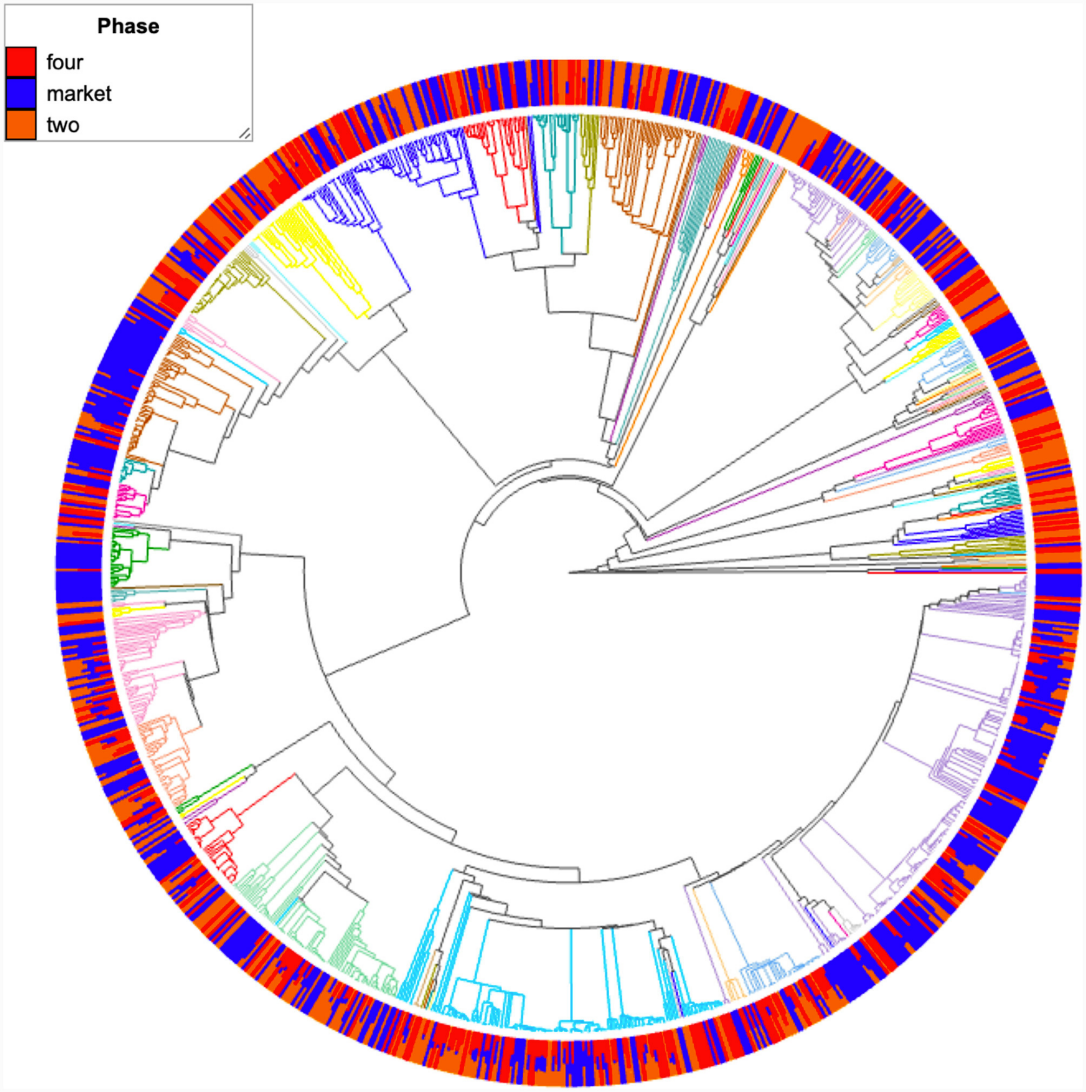
Phylogenetic tree derived from 16S rRNA gene sequence data showing the position of the most abundant bacterial taxon. Color nodes in the tree are features (taxonomic annotation to the family level). Internal nodes where all descendants have the same feature metadata value are themselves considered to have that value. Bar plots are the groups (two, four, market) metadata.

Any microbe that can multiply in food to a high level (spoilage detection level; including diseases and microorganisms utilized in food fermentation) has the theoretical potential to spoil it. Most of the food rotting has been attributed largely to bacterial species from a few taxa. Among them are *Carnobacterium* spp., *Lactobacillus* spp., *Lactococcus* spp., *Leuconostoc* spp., *Pediococcus* spp., *Streptococcus* spp., *Kurthia zopfii*, and *Weissella* spp. This is determined by the qualities of the food, the microbes, and the storage circumstances (Lorenzo et al., 2018). In the current study, the fruits originated from the market only showed the presence of bacteria from the Firmicutes family, such as *Lactobacillus sakei* (1.28%), *Weissella soli* (0.6%), *Leuconostoc mesenteroides* (< 0.1%), and *Lactococcus lactis* (40.1%), while *Carnobacterium* (4.46%) was found in all samples regardless of their origin. Most lactic bacteria species convert carbohydrates like glucose into lactic acid during the fermentation process. *Carnobacterium* is frequently the dominant species in the microflora of chilled vacuum- or modified atmosphere-packed meat, seafood, and dairy products (Laursen et al., 2005). Due to their tolerance to freezing temperatures and thawing, these species can either act as food spoilage bacteria when inappropriate storage is discovered or as protective cultures, depending on the strain and food product (Barakat et al., 2000). *Lactococcus raffinolactis* (0.4%) and *Lactococcus piscium* (< 0.1%) of the *Lactococcus* genus were detected in the fruits originating from the market only. Some strains, primarily *L. lactis*, are frequently used as starter cultures, probiotics, and protective cultures in industrial processes (Sarika et al., 2012). The Food and Drug Administration (USA) typically recognizes *L. lactis* as safe, and it is appropriate for procedures that rely on a qualified assumption of safety (EFSA, 2011). Nonetheless, *L. raffinolactis* and *L. piscium* have received attention recently as potential infections of aquaculture species (Boucher et al., 2003; Matamoros et al., 2009). At this point, we cannot appreciate the effect of these species on strawberries; further analyses are required to evaluate the effect of certain lactic bacteria on fruit quality. Using shotgun metagenomic sequencing of some exotic fruits (papaya, passion fruits), several lactic acid bacteria at the ripe stage were detected, suggesting that these bacteria might contribute to fruit fermentation (Vermote et al., 2022). From an application standpoint, this might be an advantage to obtaining fermented products to deal with excess fruit during harvest times. According to the abundance information of the main genera and species in all the samples, heatmaps and hierarchical clustering with the unweighted pair group method (UPGMA with Euclidean distance) were drawn to better visualize the change in community composition (Supplementary Figure 2). Several clusters were observed on the heat map, with samples from the market group (green color along the horizontal direction) being more similar. When comparing all eighteen samples, the hierarchical clustering analysis of phylogenetic distance in bacterial communities revealed negligible differences in the community structure in samples from the field. In addition, to reveal evolutionary relationships and biological diversity in a sequence data set, a phylogenetic tree from ASVs was built (Figure 7). The present research profiles the structure of the bacterial community in strawberries, has significantly improved our comprehension of the microbial dominance of ecosystems, and has identified several microbial species with potential technological uses.

### Fruit origin was related to quality characteristics

Berries have received particular attention in recent years because of their high concentration of potentially bioactive compounds (polyphenols, vitamins). Strawberries rank among the most popular fruits consumed worldwide (Parra-Palma et al., 2020). Fruit quality encompasses a wide range of characteristics, such as the fruit's sensory attributes (texture, appearance, skin color, sweetness, and scent), shelf life, processing attributes, resistance to pre- and postharvest contamination, and nutritional values (Parra-Palma et al., 2020). Fruit quality is influenced by phenolic compounds, which also contribute to their organoleptic and sensory qualities. In this study, functional traits (TPC, AAC, and AOX) and physicochemical parameters (pH, TTA, and TSS) were assessed in all samples. These characteristics varied throughout the groups and were strongly associated to fruit origin and at the lesser extend to fruit ripe stage (Supplementary Figure 3). The F1 component explained 69.2% of the total variance being loaded in the positive (+) direction with pH, TSS, AAC, and TPC, while the F2 component explained 23.7% of the variance being loaded in the negative (–) direction with AOX and TTA. However, the fruits collected from the field at phase two (unripe) were distinguished by acidity, group four (field) by antioxidant capacity, and the market group was defined by high acid ascorbic content and polyphenols. We observed that the variables pH and TSS were close together with a value of 0.81, indicating a high correlation (Pearson) between these cutoff values throughout the experiment. AAC and TPC showed a strong correlation (value of 0.97), as did pH and AOX (value of 0.91). A negative correlation of −0.86 was found between TTA and TSS, suggesting that as acidity raised, the total soluble solids decreased. At last, the inverse correlation between the variables AOX and TPC was found (−0.64), indicating that an increase in AOX would typically result in a decrease in TPC. Previous studies indicated that the polyphenolic content varies with the growth condition and cultivar (Aaby et al., 2012). Nonetheless, we found that the TPC was higher in commercial strawberries; these results agreed with previous research (Scalzo et al., 2005). Although we do not directly correlate the quality attributes with bacterial diversity, the results indicated a clear separation between groups according with their origin (field vs. market). Thus, we appreciate that the storage environment might contribute to bacterial colonization and invasion by other harmful and non-harmful species at the selling points. Thus, the overall quality of ready-to-eat fruits is highly influenced by the storage microclimate conditions.

## Conclusions

This is the first report on the bacterial community profile of strawberry fruits (*Fragaria* × *ananassa* variety “Monterey”) in the transition from field settings to retail market stands. A broad range of bacterial taxa have been found to inhabit strawberry fruits at both unripe and ripe stage. Our findings revealed that the number of observed ASVs, Shannon diversity, and evenness were all significantly higher in the market samples. As shown by the alpha and beta diversity analysis, the study indicates that the diversity and composition of bacterial communities differ considerably depending on their collection origin (field vs. market). While some of them have not yet been documented on this fruit, others are known to be residents. Numerous phytopathogens, including *R. bruchi, T. terrea*, and *E. rhapontici*, were found in fruits from the market and, to a lesser extent, in fruits that were collected from the field. In addition, *F. aurantia* and *E. gallinarum* were found in the market-derived samples. Discoveries of fish pathogens, *S. putrefaciens* and *S. profunda*, in two market stands raise the possibility that fruit contamination may have originated from the use of river water for irrigation. An increase in potentially pathogenic taxa on the fruit microbiome originated from the market stands is an indicator of fruit contamination postharvest. Even though metagenomic research offers a thorough picture of the diversity of bacteria in strawberry fruits from farm to table and has revealed previously unidentified pathogen species, we still need to improve our ability to decode these data and employ these knowledge to fruit safety procedures. Further work may involve the development of efficient protocols to protect the fruits postharvest to diminish both emerging pathogenic bacteria and fungi contamination postharvest.

## Data availability statement

The datasets presented in this study can be found in online repositories. The names of the repository and accession number can be found below: https://www.ncbi.nlm.nih.gov/bioproject/PRJNA1039505.

## Author contributions

GNT: Conceptualization, Data curation, Formal analysis, Funding acquisition, Investigation, Methodology, Project administration, Software, Supervision, Validation, Writing – original draft, Writing – review & editing. PR: Formal analysis, Investigation, Software, Writing – review & editing.
