# Supplementary material for: Bacterial community changes in strawberry fruits (Fragaria × ananassa variety “Monterey”) from farm field to retail market stands, an indicator of postharvest contamination
Source: Front Microbiol. 2024 Feb 16;15:1348316. doi: 10.3389/fmicb.2024.1348316 (PMC10904649; doi:10.3389/fmicb.2024.1348316)
Supplement: Supplementary file 1 [file Data_Sheet_1.docx]

Supplementary Material

Bacterial community changes in strawberry fruits (*Fragaria x ananassa* variety 'Monterey') from farm field to retail market stands, an indicator of postharvest contamination.

Gabriela N. Tenea^1^*, Pamela Reyes^1^

^1^Biofood and Nutraceutics Research and Development Group. Faculty of Engineering in Agricultural and Environmental Sciences, Universidad Técnica del Norte, 100150 Ibarra, Ecuador.

*** Correspondence:**Corresponding Author: Gabriela N. Tenea
gntenea@utn.edu.ec

# Supplementary Tables

**Supplementary Table 1.** Filtered reads of each sample upon employment of DADA2 statistics.

| **Sample ID** | **Input reads** | **Filtered reads** | **Input passed filter (%)** | **Denoised** | **Merged** | **Input merged (%)** | **Non-chimeric reads** | **Input non-chimeric reads (%)** |
| --- | --- | --- | --- | --- | --- | --- | --- | --- |
| F2L1 | 178538 | 121858 | 68.25 | 121158 | 119211 | 66.77 | 108643 | 60.85 |
| F2L2 | 214642 | 151760 | 70.70 | 151146 | 148687 | 69.27 | 137660 | 64.13 |
| F2L3 | 255442 | 178598 | 69.92 | 178035 | 175982 | 68.89 | 161627 | 63.27 |
| F2L4 | 193295 | 135886 | 70.30 | 135478 | 134023 | 69.34 | 126432 | 65.41 |
| F2L5 | 230500 | 170336 | 73.90 | 169928 | 168377 | 73.05 | 157033 | 68.13 |
| F2L6 | 214218 | 155192 | 72.45 | 154692 | 153347 | 71.58 | 144945 | 67.66 |
| F4FL1 | 167984 | 116075 | 69.10 | 115796 | 114645 | 68.25 | 102942 | 61.28 |
| F4FL2 | 136502 | 97543 | 71.46 | 97351 | 96556 | 70.74 | 90508 | 66.31 |
| F4FL3 | 157202 | 111193 | 70.73 | 110984 | 110267 | 70.14 | 105951 | 67.40 |
| F4FL4 | 248952 | 173830 | 69.82 | 173318 | 171746 | 68.99 | 155305 | 62.38 |
| F4FL5 | 209948 | 147701 | 70.35 | 147367 | 145673 | 69.39 | 140674 | 67.00 |
| F4FL6 | 191060 | 125910 | 65.90 | 125321 | 123711 | 64.75 | 118178 | 61.85 |
| FP1 | 134038 | 80957 | 60.40 | 80569 | 79335 | 59.19 | 76428 | 57.02 |
| FP2 | 126106 | 76126 | 60.37 | 75809 | 74929 | 59.42 | 71147 | 56.42 |
| FP3 | 223778 | 142398 | 63.63 | 142067 | 140338 | 62.71 | 110743 | 49.49 |
| FP4 | 255845 | 171927 | 67.20 | 171395 | 169043 | 66.07 | 150194 | 58.71 |
| FP5 | 292986 | 196126 | 66.94 | 195406 | 193199 | 65.94 | 177833 | 60.70 |
| FP6 | 344623 | 219344 | 63.65 | 218351 | 214745 | 62.31 | 202471 | 58.75 |

Legend: F2L1-F2L6: fruits collected from the agricultural field at breaking (white) ripe phase 2; F4L1-F4L6-fruits collected from the agricultural field, ripe phase 4; FP1-FP6: fruits purchased from the market, ripe phase 4.

**Supplementary Table 2.** Comparison of alpha-diversity in strawberries based on Shannon index.

| **Group 1** | **Group 2** | **H*** | **p-value** | **q-value** |
| --- | --- | --- | --- | --- |
| Four (field) | Market | 7.410 | 0.006 | 0.019 |
| Four (field) | Two (field) | 0.231 | 0.631 | 0.631 |
| Market | Two (field) | 5.026 | 0.025 | 0.037 |

*H value was compared with the critical values of the chi-square (χ²) distribution for k − 1 degrees of freedom, where k is the number of groups. If the calculated value of H is less than the critical chi-square value, there is insufficient evidence to reject the null hypothesis. This implies that there are no significant differences between the medians of the groups. If the calculated value of H is greater than the critical chi-square value, it means that there is sufficient evidence to reject the null hypothesis. This suggests that at least one of the groups has a median significantly different from the others.

**Supplementary Table 3.** Percentile abundances of features by group (ANCOM analysis).

| **Percentile** | **0** | **25** | **50** | **75** | **100** | **0** | **25** | **50** | **75** | **100** | **0** | **25** | **50** | **75** | **100** |
| --- | --- | --- | --- | --- | --- | --- | --- | --- | --- | --- | --- | --- | --- | --- | --- |
| Group | four | Four | four | four | Four | market | market | market | market | market | two | two | two | two | two |
| *Frateuria* | 1 | 1 | 1 | 1 | 1 | 121 | 243.75 | 567.5 | 1101.25 | 12040 | 1 | 1 | 1 | 1 | 1 |
| *Gluconobacter* | 1 | 1 | 1 | 1 | 7 | 50 | 452.25 | 1794 | 2553 | 7185 | 1 | 1 | 1 | 1 | 25 |
| *Yersinia* | 1 | 1 | 1 | 1 | 1 | 76 | 137.75 | 242 | 436.25 | 9730 | 1 | 1 | 1 | 1 | 83 |
| *Lactococcus* | 1 | 1 | 1 | 1 | 120 | 112 | 387 | 624.5 | 4627.75 | 61473 | 1 | 1 | 2.5 | 4 | 135 |
| *Lactobacillus* | 1 | 1 | 1 | 1 | 1 | 3 | 51.75 | 91 | 1692.5 | 3151 | 1 | 1 | 1 | 1 | 1 |

**Supplementary Figure 1.** Data analysis workflow.

**
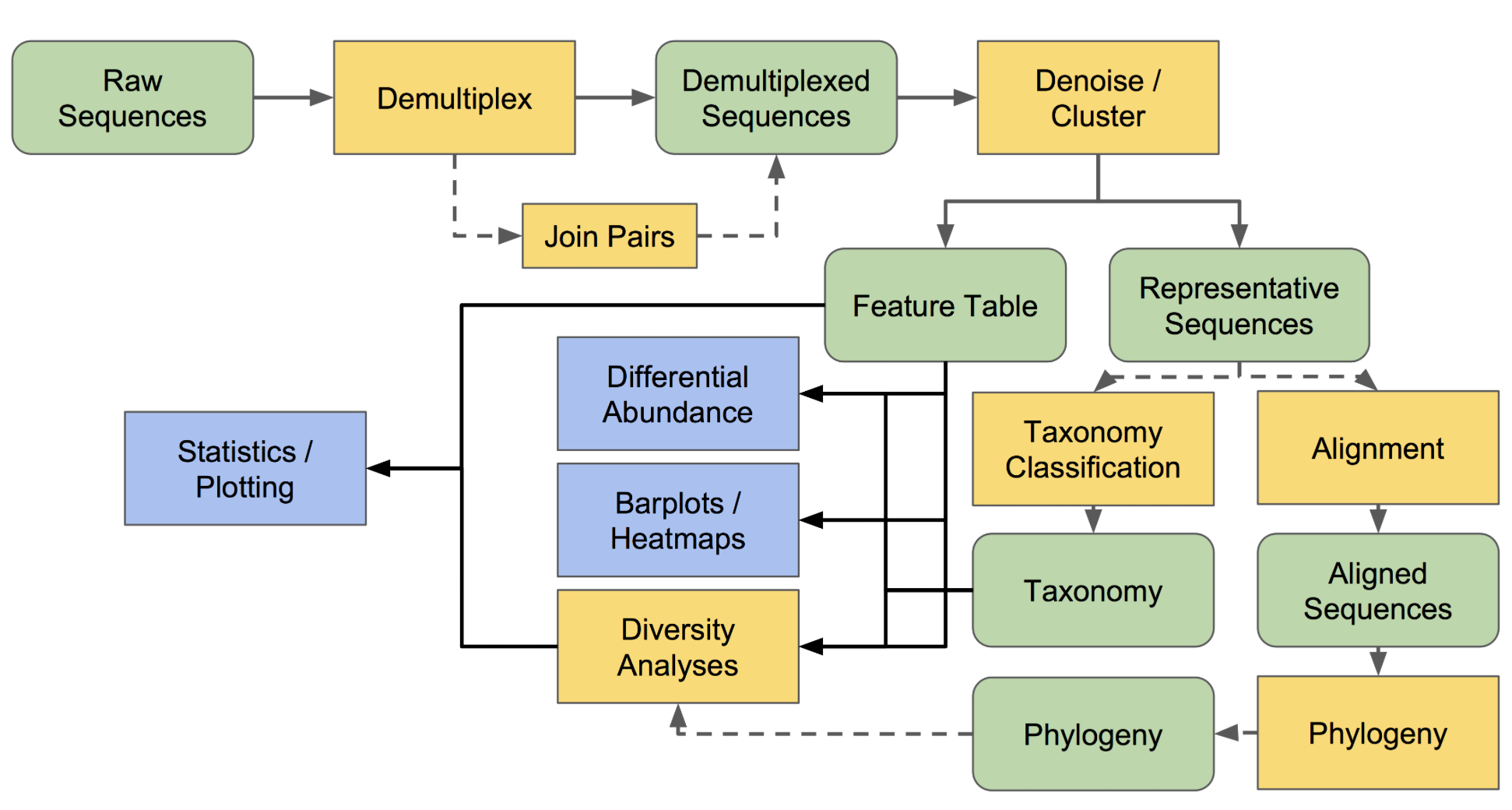
**

**Supplementary Figure 2.** Heatmap and hierarchical clustering of the most abundant bacterial taxa. X-axis contains the microbial taxa that have been identified in the samples; On the Y axis are the different samples and experimental conditions that are being compared (groups). The colors in the heat map represent the relative abundance of each microbial taxon in each sample, warmer colors represent a high abundance of these microorganisms in a sample, while darker colors indicate low abundance. Color bars: green: samples from the market (phase 4); Blue: samples from the field, phase 4; orange: samples from the field phase 2.


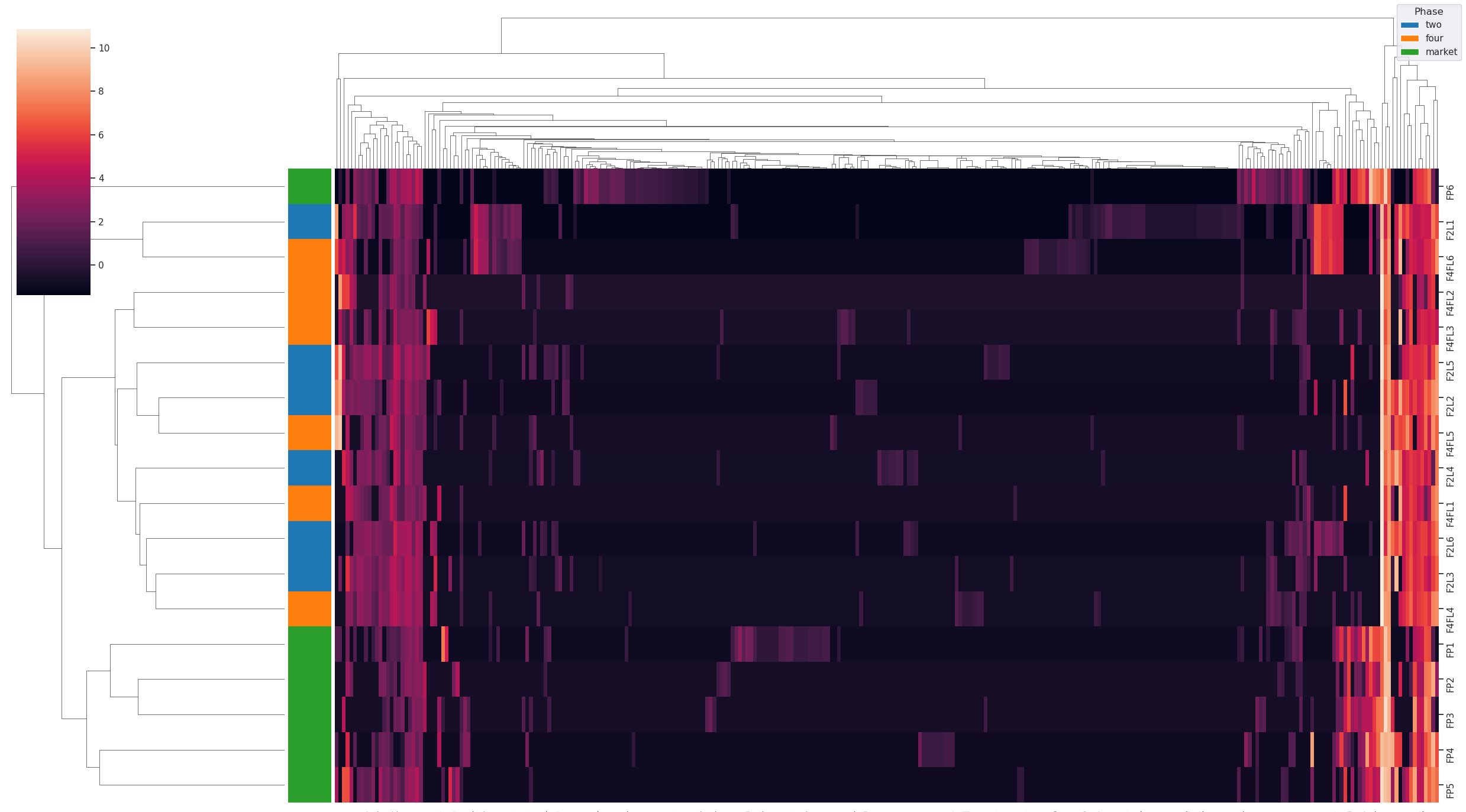


**Supplementary Figure 3.** Biplot PCA analysis of the six variables (pH, total titratable acidity-TTA, total soluble solids-TSS, antioxidant capacity-AOX, acid ascorbic content-AAC, and total polyphenol content-TPC) of strawberries. The colors marked the close-related samples registered for each variable. F2L1-F2L6: fruits collected from agricultural field phase 2; F4L1-F4L6-fruits collected from agricultural field phase 4; FP1-FP6: fruits purchased from market phase 4-5.
